# Comparing the efficacy of teriparatide and bisphosphonates in osteoporotic patients undergoing thoracolumbar spinal fusion: a meta-analysis

**DOI:** 10.3389/fsurg.2025.1656134

**Published:** 2025-10-21

**Authors:** Xinghua Ji, Jinhui Duan, Guoyu He, Junjie Wang, Zejun Xing

**Affiliations:** 1 Shanxi Bethune Hospital, Shanxi Academy of Medical Sciences, Tongji Shanxi Hospital, Third Hospital of Shanxi Medical University, Taiyuan, China; 2 Tongji Hospital, Tongji Medical College, Huazhong University of Science and Technology, Wuhan, China; 3 Department of Orthopaedic Surgery, Shengjing Hospital of China Medical University, Shenyang, China

**Keywords:** teriparatide, bisphosphonate, thoracolumbar spinal fusion, meta-analysis, osteoporosis

## Abstract

**Objectives:**

This study compared the efficacy of teriparatide and bisphosphonates(BPs) in osteoporotic patients who underwent thoracolumbar spinal fusion through a comprehensive meta-analysis of published data from randomized controlled trials and other types of comparative studies.

**Methods:**

Major online databases, including PubMed, EMBASE, Web of Science, and Cochrane Central, were searched for studies on the efficacy of teriparatide and BPs in patients treated with thoracolumbar spinal fusion. Studies that evaluated bone union conditions and other subjective or objective outcomes were included. The publication period ranged from database inception to June 13, 2025.

**Results:**

Ultimately, 6 articles with 329 patients were included in this meta-analysis. Compared with BPs, teriparatide had advantages in terms of improving the bone union rate at both the 6-month and 1-year follow-ups and reducing the Oswestry Disability Index (ODI) and leg pain visual analogue scale (VAS) score at the 1-year follow-up. The screw loosening rate, VAS score for low back pain at the 1-year follow-up and ODI score at the 6-month follow-up were not clearly different between the teriparatide and BP groups.

**Conclusion:**

In patients undergoing thoracolumbar spinal fusion, compared with BPs, teriparatide clearly promotes bone fusion and improves quality of life, with high safety. However, the small number of studies included in this meta-analysis (six) may have influenced the results in the forest plots.

## 1 Introduction

Spinal fusion surgery for terminal thoracic spine, lumbar spine and initial sacral spine yields similar outcomes compared to surgery for cervical vertebrae. These fusion surgeries, such as Posterior lumbar interbody fusion and transforaminal lumbar interbody fusion, are the most common surgical methods for lumbar degenerative diseases and spinal fractures (1). These surgeries can relieve nerve compression, thereby alleviating pain and neurological symptoms (2, 3). The final outcome of spinal fusion is a combination of bone formation and remodelling, resulting from both bone formation and resorption (4). However, lumbar degenerative diseases and spinal fractures are more common in older individuals (5, 6); as people age, they typically suffer from osteoporosis, another common disease (7, 8) that is characterized by an imbalance between osteoblasts and osteoclasts that results in a fragile bone microstructure and a low bone mineral density (BMD). Patients with osteoporosis who undergo spinal fusion surgery tend to experience more complications, such as fusion failure, cage subsidence and vertebral compressive fractures (VCFs) (8, 9). Therefore, the management of osteoporosis may improve the outcomes of spinal fusion surgery.

Antiosteoporosis drugs can be classified according to their functions, including those that inhibit bone resorption, such as bisphosphonates (BPs) and denosumab; those that promote bone anabolism, such as teriparatide; and those that influence both functions, such as romosozumab (10). Among these antiosteoporotic drugs, BPs, one of the earliest and most classical group of drugs, are recommended as the first-line medication by most researchers and physicians, who recommend that patients with osteoporosis receive BP treatment to control their decreasing BMD (11, 12). However, one previous meta-analysis suggested that although BPs can clearly reduce the rates of vertebral compression fracture, cage subsidence, and pedicle screw loosening after spinal fusion, they do not greatly increase the bone fusion rate (13). Compared with BPs, which inhibit the activity of osteoclasts, teriparatide can clearly induce the activity of osteoblasts and promote bone formation since it is a recombinant form of a human parathyroid hormone fragment (14), which presents similar efficacy in thoracolumbar spines (15). One previous meta-analysis documented the efficacy of teriparatide in improving the bone fusion rate after spinal fusion surgery (16). The difference in the clinical efficacy between BPs and teriparatide may be due to their different effects on bone metabolism; therefore, the corresponding literature should be summarized to obtain greater insights into these differences and aid in developing more efficient drugs. From 2018 to 2020, previous systematic and meta-analyses compared the efficacy of teriparatide and BPs in patients undergoing thoracolumbar spinal fusion (17–19); however, some of the meta-analyses included studies that compared one drug with a control group. In addition, several studies in this field have been published since the publication of the two meta-analyses. Therefore, performing an updated meta-analysis to compare these two drugs directly and provide information on evidence-based medicine to clinical doctors when making clinical decisions is necessary.

This study compared the efficacy of teriparatide and BPs in osteoporotic patients who underwent thoracolumbar spinal fusion by performing a comprehensive meta-analysis of published data from randomized controlled trials (RCTs) and other types of comparative studies.

## 2 Material and methods

### 2.1 Search strategy

Two independent researchers screened major online databases, including PubMed, EMBASE, Web of Science, and Cochrane Central, with a publication period ranging from database inception to June 13, 2025. The key terms used for searching the titles and abstracts were “interbody fusion”, “lumbar fusion”, “spinal fusion”, “thoracic vertebra”, “teriparatide”, “bisphosphonate”, “alendronate”, “clodronate”, “etidronate”, “ibandronate”, “minodronate”, “neridronate”, “olpadronate”, “pamidronate”, “risedronate”, “tiludronic acid”, and “zoledronic acid”; these terms were used in different combinations.

### 2.2 Eligibility criteria and study identification

Articles were included if they met the following criteria: (1) target population—osteoporosis patients who suffered from thoracolumbar spinal disease; (2) intervention—spinal interbody fusion, with one group using teriparatide and the other group using BPs; (3) outcomes—parameters evaluating BMD, the bone union rate and other subjective or objective outcomes; (4) type of study—although RCTs were desirable, other types of comparative studies were also accepted; and (5) language—English.

Using the inclusion criteria listed above, the two researchers independently read the titles and abstracts in the online databases. If they thought the article was eligible, they read the full text. Any disagreements were resolved by discussion with a third investigator.

### 2.3 Data extraction and risk of bias assessment

After identifying the included studies, the two researchers extracted the following information from the eligible articles: first author names, publication year, the number of patients allocated to each group, the number of male patients in each group, the mean body mass index (BMI) of each group, the mean age of each group, major diagnosis, surgery type, the intervention method used in each group, and treatment and follow-up durations. The quality of the RCTs was assessed using the Cochrane risk of bias tool (20), whereas the Newcastle–Ottawa Scale (NOS) was used to assess the quality of other types of comparative studies (21).

### 2.5 Statistical analysis

RevMan 5.3 software was used to conduct the statistical analyses. The odds ratio (OR) and 95% confidence interval (CI) were computed as summary statistics for the dichotomous variables, and pooled summary statistics were calculated with the use of a random effects model. The mean difference (MD) and 95% CI were computed as summary statistics for continuous variables, and pooled summary statistics were calculated with the use of a fixed effects model if the heterogeneity was not significant; otherwise, a random effects model was applied. *P* < 0.05 was regarded as statistically significant. Statistical heterogeneity was quantified using chi-square (*χ*^2^) and I^2^ tests, and heterogeneity was considered to exist based on *P* < 0.05 or *I*^2^ > 50%. Getdata (version 2.22) was used if the data were presented only as figures in the article instead of as detailed information.

## 3 Results

### 3.1 Literature search

**Figure 1:**
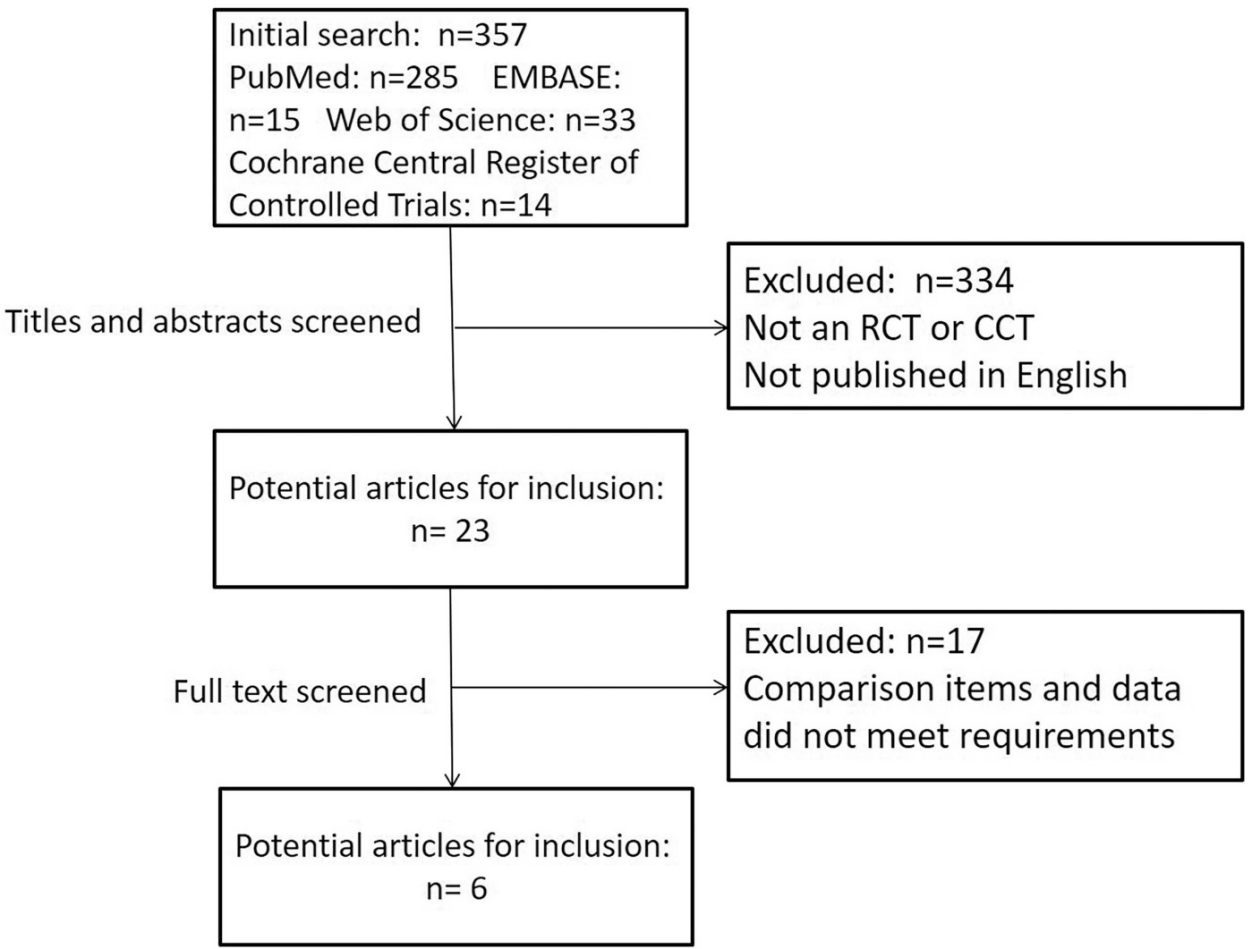
Flow diagram of the eligible studies included in this meta-analysis.

After eliminating duplicate articles, 357 studies identified from the electronic databases using the search strategy listed above were included. Of these studies, 285 were from PubMed, 15 were from EMBASE, 33 were from Web of Science, and 14 were from Cochrane Central. A total of 334 studies were then excluded since they were not RCTs or comparative cohort trials (CCTs) or were not published in English. After the full texts were read, 15 studies were found to have data that could not be included in the meta-analysis. Two studies involved a mixed intervention of teriparatide and BPs. Ultimately, 6 articles were included in this meta-analysis (22–27). Figure 1 presents the process of screening and identifying eligible studies.

### 3.2 Study characteristics

The main characteristics of the 6 included studies are summarized in Table 1. Only one study was an RCT. The two studies published after 2020 were conducted in China, while the other four studies were conducted in Japan. Participants in the teriparatide group of one study were treated with teriparatide for an average of 13 months and then with BPs until 15 months after surgery; however, some parameters were evaluated 12 months after surgery; therefore, the RCT was included in this study, and a meta-analysis was conducted for the outcomes that were measured at the 12-month follow-up (26). The publication period ranged from 2012 to 2022. A total of 329 patients were analysed in this work. One study included 7 male patients, and only two studies provided detailed information on BMI. The two most recent studies used zoledronic acid, whereas the other four studies used oral BPs. The duration of the intervention ranged from 1 to 2 years.

**Table 1:** Characteristics of the included studies.

First author	Publication year	Type of study	Number of patients in the teriparatide/BP groups at baseline	Number of male patients in the teriparatide/BP groups at baseline	Age of patients in the teriparatide/BP groups at baseline	BMI of patients in the teriparatide/BP groups at baseline	Major diagnosis	Type of surgery	Intervention methods in the teriparatide/BP groups	Preoperative BMD in the teriparatide/BP groups	Treatment duration	Follow-up duration after surgery
Ohtori et al. (24)	(2012)	Prospective study	29/28	0/0	76 ± SD 6.5 (range 62–84)/77 ± SD 6.0 (range 53–83)	Not reported	Women with osteoporosis diagnosed with degenerative spondylolisthesis	Instrumented lumbar posterolateral fusion using local bone grafting	Daily subcutaneous injection of 20 μg of teriparatide/weekly oral administration of 17.5 mg of risedronate (2 months before surgery)	Young adults mean(%):65 ± SD 5 (range 58–70)/64 ± SD 6 (range 59–70)	2 months before and 8 months after surgery	1 year
Ohtori et al. (27)	(2013)	Prospective study	20/20	0/0	78 ± SD 6.0 (range 60–82)/75 ± SD 5.0 (range 55–84)	Not reported	Women with osteoporosis diagnosed with degenerative spondylolisthesis	1- or 2-level instrumented posterolateral fusion with a local bone graft	Daily subcutaneous injection of 20 μg of teriparatide/daily oral administration 2.5 mg of risedronate(2 months before surgery)	Young adults mean(%):66 ± SD 4 (range 58–69)/64 ± SD 4 (range 58–71)	2 months before and 10 months after surgery	1 year
Ohtori et al. (26)	(2015)	Retrospective case series	15/15	0/0	72 ± SD 7.7 (range 53–82)/71 ± SD 6.0 (range 58–84)	Not reported	Women with osteoporosis diagnosed with degenerative spondylolisthesis	Lumbar posterolateral fusion using local bone grafting	Daily subcutaneous injection of 21 µg of teriparatide/weekly oral administration of 17.5 mg of risedronate	Young adults mean(%):65.6 ± SD 6 (range 62–70)/66.8 ± SD 8.0 (range 55–70)	3 months before and 13 months after surgery for each group, and then transferred to BPs until 15 months after surgery	15 months
Seki et al. (22)	(2017)	Prospective study	25/33	0	72.5 ± SD 5 (range 60–81)/71.5 ± SD 2 (range 58–83)	Not reported	Japanese women with osteoporosis diagnosed with adult spinal deformity	Posterior facet joints and laminar fusions	Single self-administered subcutaneous injection of 20lg of teriparatide per day/alendronate at 5 mg/day (5 patients) or risedronate at 2.5 mg/day (20 patients)	0.652 ± SD 0.128 g/cm2 (T- score −3.9 ± SD 0.2)/0.667 ± SD 0.118 g/cm2 (T-score −3.8 ± SD 0.2)	3 months before and 21 months after surgery	2 years
Wang et al. (25)	(2021)	Retrospective cohort study	29/38	4/3	66.34 ± SD 8.13/65.89 ± SD 6.13	23.20 ±SD 3.21/24.10 ± SD 3.75	Osteoporosis patients with lumbar disc herniation, lumbar spinal stenosis, scoliosis, degenerative lumbar instability, or spondylolisthesis	Transforaminal lumbar interbody fusion	Teriparatide (20 μg/day, once daily) subcutaneously and continuously for more than 6 months starting 1 day after surgery/zoledronic acid once intravenously for at least 15 min (5 mg/year) to 3 days after surgery	0.712 ± SD 0.138 g/cm2/0.730 ± SD 0.123 g/cm2		12 months
Xiong et al. (23)	(2022)	RCT	36/41	0/0	63.1 ± SD 7.4/63.6 ± SD 8.3	24.3 ± SD 3.1/24.6 ± SD 3.3	Postmenopausal women with osteoporosis and degenerative spondylolisthesis	Single level posterior lumbar interbody fusion	Daily subcutaneous injections of 80 ml (20 mg) of teriparatide/intravenous infusion of 5 mg of zoledronic acid diluted in 100 ml of saline, once annually for 2 years, antiosteoporosis treatment was initiated 3 days after surgery	0.623 ± SD 0.041/0.631 ± SD 0.042		2 years

### 3.3 Study quality

**Figure 2:**
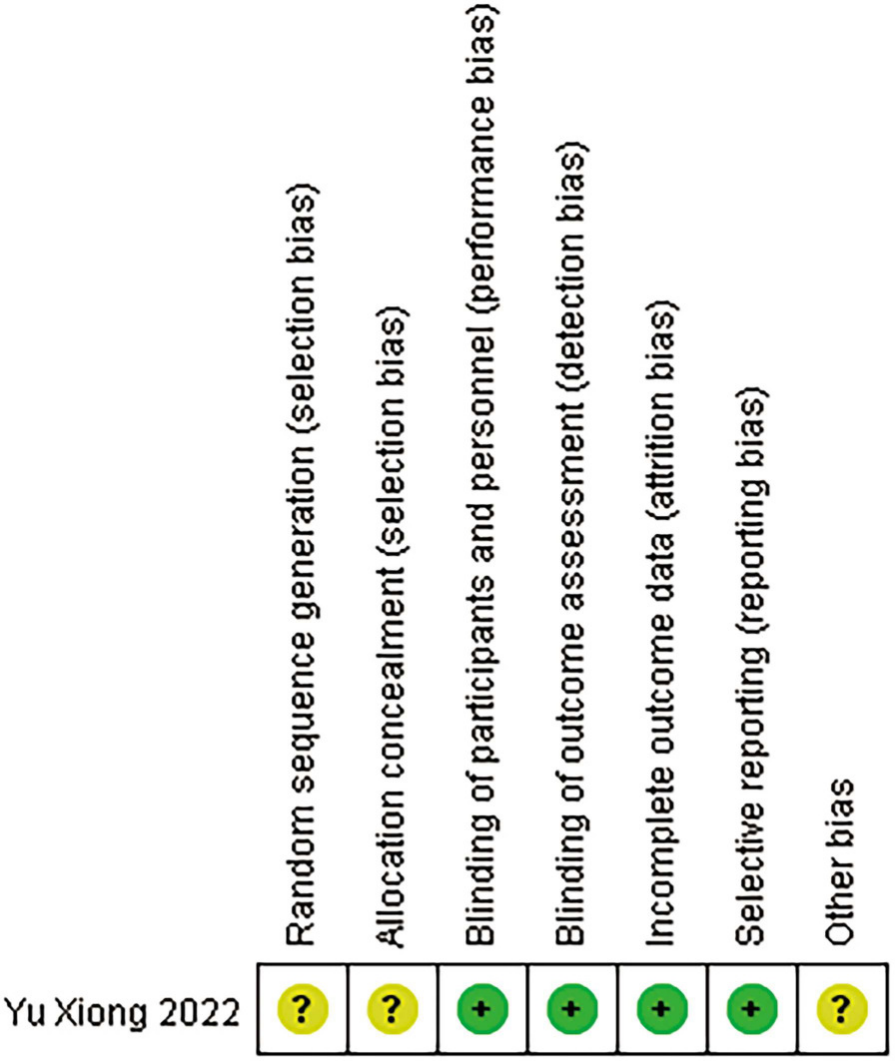
Quality of the included RCTs.

The six included studies were of relatively high quality (Figure 2). The RCT was considered to have a low risk of bias in terms of the blinding methods, the completeness of the outcome data and selective reporting. The CCTs were of good quality, with 4 of the 5 CCTs receiving a score of 7 on the NOS and the last study receiving a score of 5 (Table 2).

**Table 2:** Quality of the included cohort trials.

NOS scoring terms		Ohtori et al. (24)	Ohtori et al. (27)	Ohtori et al. (26)	Seki et al. (22)	Wang (25)
Selection						
	Representativeness of the exposed cohort	*	*	*	*	*
	Ascertainment of exposure	*	*		*	*
	Outcome not present at the start of the study					
Comparability						
	Comorbidities	*	*	*	*	*
	Other factors	*	*	*	*	*
Outcome						*
	Assessment of the outcome	*	*		*	*
	Follow-up period long enough for the outcome to occur	*	*	*	*	*
	Adequacy of the follow-up	*	*	*	*	*
Total		7	7	5	7	7

*One point.

According to Egger et al. (28), an assessment of publication bias comparing fewer than 10 studies is not reliable. Therefore, a funnel plot was not constructed to evaluate publication bias.

### 3.4 Bone union condition

**Figure 3:**
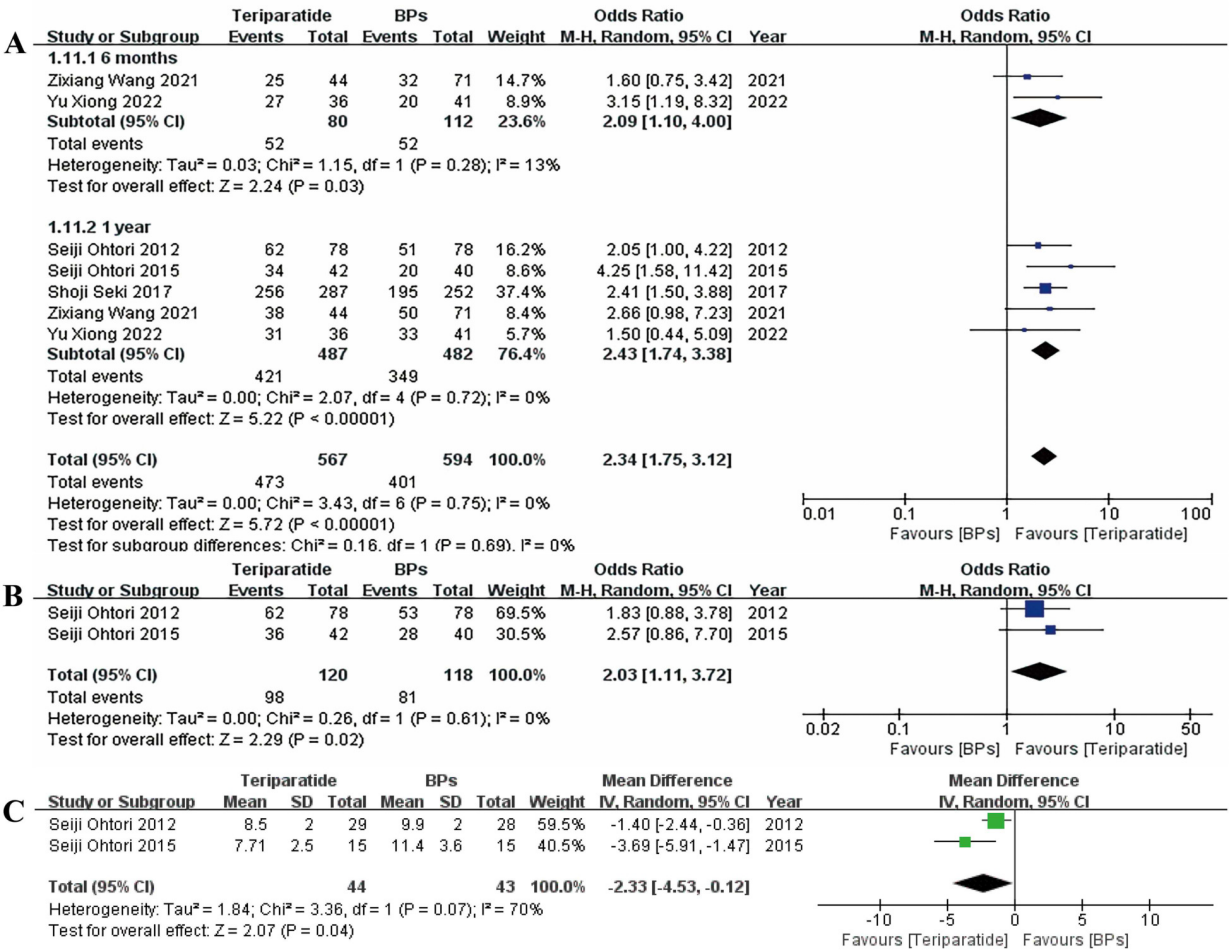
Forest plots of **A** the bone union rate evaluated by CT at the 6-month and 1-year follow-ups, **B** the bone union rate evaluated by radiology at the 1-year follow-up, and **C** the bone union period.

Figure 3 shows the results for the bone union rate evaluated by computed tomography (CT) (the number of facet joints) at the 6-month and 1-year follow-ups, the bone union rate assessed by radiology (the number of segments) at the 1-year follow-up, and the bone union period. The forest plots suggest that, compared with the BP group, the teriparatide group had an obviously shorter bone union period and a higher bone union rate at both the 6-month and 1-year follow-ups. A random-effects model was applied to generate the forest plot for bone union period, since this is a continuous variable and the associated heterogeneity was high (*I*^2^ > 50%), while the forest plots for bone union rate were obtained with a random-effects model since it was a dichotomous variable.

### 3.5 Screw loosening rate

**Figure 4:**
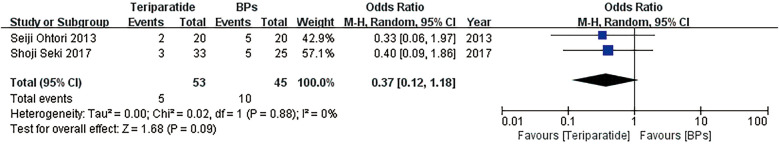
Forest plot of the screw loosening rate.

Figure 4 presents forest plots of the screw loosening rate at the 1-year follow-up, and the results revealed no significant difference between teriparatide and BPs. The forest plot was obtained with a random-effects model since this was a dichotomous variable.

### 3.6 Patient-reported outcomes

**Figure 5:**
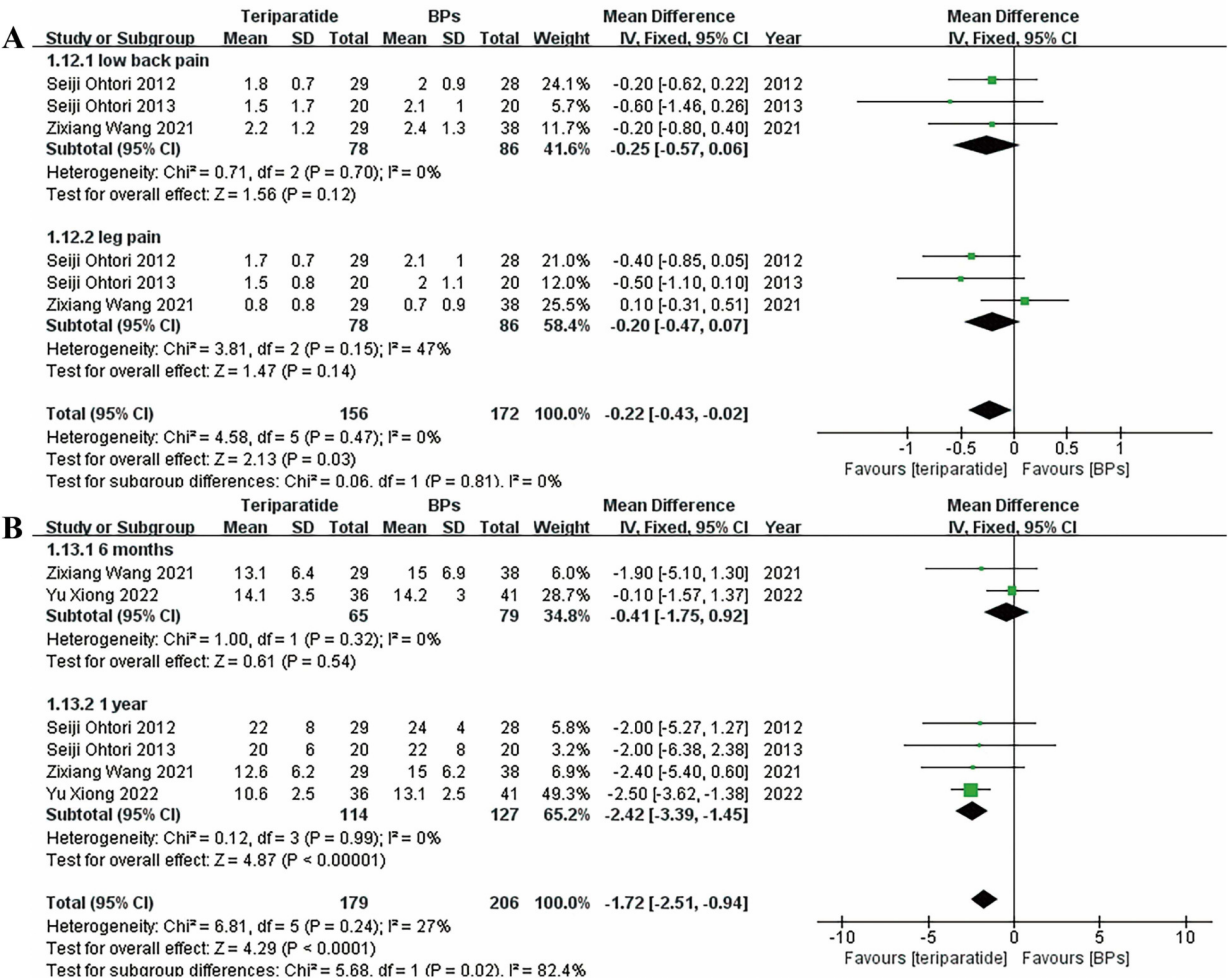
Forest plots of **(A)** the VAS score for low back pain and leg pain at the 1-year follow-up, and **(B)** the ODI score at the 6-month and 1-year follow-ups.

Figure 5 presents the forest plots of the visual analogue scale (VAS) scores for low back pain and leg pain at the 1-year follow-up and the Oswestry Disability Index (ODI) scores at the 6-month and 1-year follow-ups, respectively; the results suggested no significant differences in the ODI score at the 6-month follow-up or the VAS score at the 1-year follow-up between the two groups. However, teriparatide could greatly lower the ODI score at the 1-year follow-up compared with BPs. A fixed-effects model was used to generate the forest plots of the VAS score and ODI since they were continuous variables and the associated heterogeneity was low (*I*^2^ < 50%).

### 3.7 Sensitivity analysis

**Figure 6:**
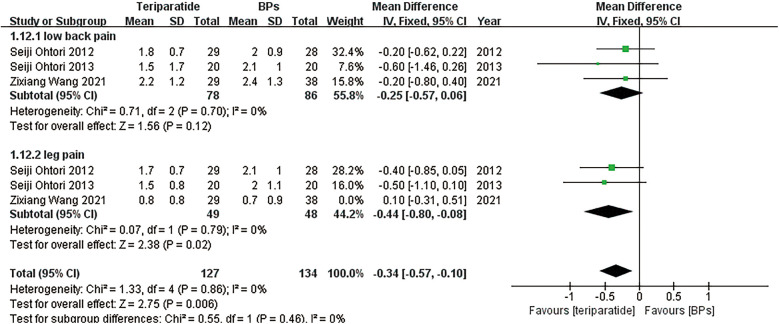
Sensitivity analysis.

Although most comparisons presented low heterogeneity, the heterogeneity of the forest plot for the bone union period was high; nevertheless, sensitivity or subgroup analyses could not be conducted since only two studies were included in this comparison. In the comparison of the VAS score of leg pain, the heterogeneity was moderately high (*I*^2^ = 47%); to reduce the heterogeneity, the study conducted by Zixiang Wang et al. was omitted since, unlike the other two studies, which used risedronate, they used zoledronic acid. The results noticeably changed with the removal of this study (Figure 6), indicating that the teriparatide group had a significantly lower VAS score than the BPs group.

## 4 Discussion

This meta-analysis summarized previously published results regarding two major areas: fusion conditions and patients' subjective feelings. Overall, the results of the forest plots presented above suggest that, compared with BPs, teriparatide has advantages in terms of improving the bone union rate at both the 6-month and 1-year follow-ups and reducing the ODI and leg pain VAS score at the 1-year follow-up. The screw loosening rate, VAS score for low back pain at the 1-year follow-up and ODI score at the 6-month follow-up were not clearly different between the teriparatide and BP groups.

Three outcomes for bone union conditions were evaluated in the meta-analysis: the bone union period, the screw loosening rate and the bone union rate. The bone union period is a new parameter that has not been compared in previous meta-analyses. Our forest plot suggested that, compared with BPs, teriparatide clearly shortened the bone union period and therefore promoted bone union, although the heterogeneity was slightly high in the comparison. The bone union rate, as evaluated by CT and radiology, also supported this result. In addition, in a study conducted by Seiji Ohtori et al. in (24) the stability rate of the teriparatide group was higher than that of the BP group, which was similar to the results of another study conducted by Seiji Ohtori et al. in (26); however, because the follow-up period of the study in 2015 covered the BP treatment period in the teriparatide group, a meta-analysis could not be conducted. No included study reported detailed information about the risks for compression fracture, VCFs and cage subsidence, which may be advantages of BPs according to previous studies (13, 17). However, although BPs have been reported to be effective in reducing screw loosening and our results also suggest no significant difference in this parameter between teriparatide and BP treatments, the trend of our forest plot is quite obvious for the teriparatide group, which had a lower loosening rate, perhaps because the small sample size affected the results. Shoji Seki et al. also suggested better outcomes for VCFs in the teriparatide group than in the BP group, but no other included studies reported this outcome; thus, a meta-analysis was unable to be conducted. Additionally, teriparatide has been shown to exhibit similar efficacy in thoracic and lumbar spine (15). However, no previous study has demonstrated the effect of BPs on the two locations. Further studies are needed to explore these topics.

Two patient-reported outcomes were included in the meta-analysis: VAS and ODI scores. The VAS is a common tool for evaluating pain, while the ODI is used to assess self-care ability in daily life; higher scores on these two tools indicate worse outcomes (29, 30). Compared with one previous study (17), we compared these parameters at each follow-up point instead of mixing the results at different follow-up points. Although the results suggested that teriparatide could reduce the ODI and leg pain VAS score only at the 1-year follow-up, the trend of the forest plots for teriparatide was clearly a reduction in the VAS score for low back pain at the 1-year follow-up and the ODI score at the 6-month follow-up; once again, the small sample size may have affected the results. In addition, Yu Xiong et al. reported no significant difference in the ODI score at the 2-year follow-up between the teriparatide and BP groups; however, Shoji Seki et al. obtained significant differences in the ODI score at the 2-year follow-up. The different diagnosis and scoring methods, as well as the BP type may have caused this difference, and further studies are needed to explore this issue. In addition, Shoji Seki et al. reported the Scoliosis Research Society questionnaire-22 score, a parameter that also evaluates the pain domain, which also suggested better outcomes for the teriparatide group than for the BP group. A meta-analysis could not be conducted since no other included studies reported this parameter.

The two most recent included studies provided detailed information on BMD (7, 23), which can reflect the direct therapeutic efficacy of the two drugs. Both studies suggested a better BMD outcome for the teriparatide group than for the BP group. Moreover, they both suggested limited efficacy for BPs in increasing BMD after spinal fusion surgery. Nevertheless, the two studies did not assess the same location; therefore, their data could not be subjected to a meta-analysis, and the number of included patients may also be small. Wang et al. also presented a change in the expression of bone turnover markers, including P1NP and β-CTX, suggesting that the levels of these two markers were increased in the teriparatide group and decreased in the BP group; it is widely accepted that the decrease in β-CTX represents a relief in bone resorption, while the increase in P1NP is a sign of active bone anabolism (31, 32). Our results are consistent with those of previous studies, reflecting the effects of each drug. Five of the six included studies reported associated adverse effects of the two drugs, except the study conducted by Xiong et al., and none of the studies reported drug-related adverse effects in either group, which suggested the safety of the two drugs. However, in women older than 65 years, a previous study suggested a relatively greater risk of adverse effects in the BP group than in the control group (33); the number of articles regarding the adverse effects of teriparatide was low, which may suggest an advantage in the safety of teriparatide over BPs.

Compared with the previous meta-analysis, this work included more studies and compared more parameters to compare the efficacy of teriparatide and BPs in patients who underwent thoracolumbar spinal fusion and obtained different results (19). Nevertheless, limitations are unavoidable. First, the major limitation of this study is the small number of included studies, despite the inclusion of CCTs to increase the number of included trials. Furthermore, the RCT included here presented no clear difference in the forest plots when conducting sensitivity analysis, as did elimination of the study that scored a 5 on NOS. This small number of studies may have decreased the reliability of the forest plots. Additionally, meta-regression analysis and funnel plots could not be used to identify the sources of heterogeneity and bias due to the small number of studies. Moreover, the small sample size may obscure potentially scarce but serious adverse effects for the two drugs. Second, the intervention methods, surgical techniques, drugs doses and BP types differed across the included studies, which may have caused heterogeneity and impaired the reliability of the forest plots. Third, the efficacy of the two drugs could not be compared because we did not have results for direct BMD parameters. Fourth, previous studies have suggested that farnesyl pyrophosphate synthase gene polymorphisms are a key mechanism in the response to BPs, which is absent in eastern Asians (34, 35), indicating that racial differences may influence the antiosteoporotic effect; therefore, racial bias may have affected the results since only eastern Asians were included in the current study. Finally, selection and publication bias may be present since only English language articles were included.

## 5 Conclusions

This meta-analysis revealed that, compared with BPs, teriparatide clearly promotes bone fusion and improves the quality of life of patients who underwent thoracolumbar spinal fusion, with high safety. However, a small number of studies (six) was included in this meta-analysis, which may have influenced the results depicted in the forest plots. Further work involving larger sample sizes should be conducted to confirm the results and provide stronger evidence in this field.

## Data Availability

The original contributions presented in the study are included in the article/Supplementary Material, further inquiries can be directed to the corresponding author.
